# Clinicopathological Characteristics and Outcomes of Chinese Patients with Scanty Immune Deposits Lupus Nephritis: A Large Cohort Study from a Single Center

**DOI:** 10.1155/2014/212597

**Published:** 2014-02-04

**Authors:** Qiuyu Li, Di Song, Fengmei Wang, Ying Tan, Feng Yu, Minghui Zhao

**Affiliations:** ^1^Renal Division, Department of Medicine, Peking University First Hospital, Beijing 100034, China; ^2^Institute of Nephrology, Peking University, Beijing 100034, China; ^3^Key Laboratory of Renal Disease, Ministry of Health of China, Beijing 100034, China; ^4^Key Laboratory of Chronic Kidney Disease Prevention and Treatment, Ministry of Education, Beijing 100034, China

## Abstract

*Objective*. To assess clinicopathological characteristics of lupus nephritis patients with scanty immune deposits. *Methods*. The data of patients with scanty immune deposits lupus nephritis were retrospectively analyzed. Plasma ANCA and complement components were detected. *Results*. Among 316 cases with renal biopsy-proven lupus nephritis, 40 cases were diagnosed as scanty immune deposits. There were significantly higher value of serum creatinine (*P* = 0.002) and lower hemoglobin level (*P* = 0.009) and higher score of cellular crescents (*P* = 0.015) in scanty immune deposits group compared with immune complex deposits group. The frequency of positive plasma ANCA was significantly higher in scanty immune deposits group than that in immune complex deposits group (52.5% versus 10.1%, *P* < 0.001). As for comparisons of plasma complement components, there were significantly higher levels of C1q (*P* = 0.005) and Bb (*P* = 0.02) and lower level of factor H (*P* = 0.003) in scanty immune deposits group. The ratio of treatment failure was significantly higher in scanty immune deposits group than that in immune deposits group (42.5% versus 19.20%, *P* = 0.001). The renal outcomes were similar between the two groups. *Conclusions*. Patients with scanty immune deposits lupus nephritis had more severe kidney damage. ANCA and activation of complement alternative pathway might be involved in the pathogenesis of the disease.

## 1. Introduction

Systemic lupus erythematosus (SLE) is a prototypic autoimmune disease characterized by the production of multiple autoantibodies. Renal involvement is common in SLE. The typical feature in lupus nephritis is immune complex deposition, showed as “full house” under immunofluorescence observation. However, in previous reports, some patients with lupus nephritis presented with “scanty immune deposits,” that is, nonclassical glomerulonephritis, which might contribute to thrombotic microangiopathy (TMA) [1], ANCA-associated crescentic glomerulonephritis [2] podocytopathy [3], and so forth. Here, the “scanty immune deposits” were indicated as a descriptive term to identify the specimens with little or no staining for immunoglobulin and not necessarily for lesions with necrosis or crescents. The clinicopathological features, outcomes, and possible pathogenesis of scanty immune deposits lupus nephritis have not been well delineated and extensively studied.

This study is to assess clinical manifestations, laboratory characteristics, pathological features, and outcomes of patients with scanty immune deposits in a large cohort of Chinese lupus nephritis patients. Particularly, we further detect the distribution of ANCA and complement activation profile in the patients.

## 2. Methods

### 2.1. Patients

Renal histopathological data of 316 patients with renal biopsy-proven lupus nephritis, diagnosed between January 2000 and July 2008 in Peking University First Hospital, were reviewed and reclassified according to the International Society of Nephrology and Renal Pathology Society (ISN/RPS) 2003 classification [4]. Only biopsy specimens with more than 10 glomeruli were included in the study.

On frozen sections of renal biopsy, at least two glomeruli, except for the sclerosed glomeruli, were evaluated by a renal pathologist. Scanty immune deposition was defined as negative staining or 1+ positivity (on a scale of 0–4+) of immunoglobulins (IgG, IgA, and IgM) by direct immunofluorescence assay and no electron-dense deposit in glomeruli, tubular basement membrane, and vessels was observed by electron microscopy assay. Immune complex deposits were defined as (i) a score of 2+ or higher in staining for any kind of immunoglobulin observed by immunofluorescence microscopy and (ii) electron-dense deposits observed by electron microscopy [5].

The patients fulfilled the 1997 American College of Rheumatology revised criteria for SLE [6].

### 2.2. Clinical Evaluation

The following clinical data were collected and analyzed: gender, fever, malar rash, photosensitivity, oral ulcer, alopecia, arthritis, serositis, neurologic disorder, anemia, leukocytopenia, thrombocytopenia, hematuria, and leukocyturia. The criteria for system involvement were consistent with the 1997 American College of Rheumatology revised criteria for SLE [6]. The clinical disease activity was assessed by the Systemic Lupus Erythematosus Disease Activity Index (SLEDAI) [7, 8].

The renal response to the therapy includes complete remission, partial remission and treatment failure was detailed in previous studies [9–12].

A relapse was defined as (1) nephritic relapse: a recent increase of serum creatinine by >50% with active urinary sediments; (2) proteinuric relapse: development of either a nephrotic syndrome (proteinuria >3.5 g/day and serum albumin <30 g/L) or proteinuria >1.5 g/day without other causes, in previously nonproteinuric patients [13, 14].

The patients were followed up in outpatient clinic specified for patients with lupus nephritis. The primary end point was defined as death and the secondary end points were defined as end-stage renal disease (ESRD) or doubling of serum creatinine.

### 2.3. Laboratory Assessment

The following laboratory features were further detected using serum or plasma at the day of renal biopsy.

Serum antinuclear antibodies (ANA) were detected using indirect immunofluorescence assay (EUROIMMUN, Lübeck, Germany) and anti-double-stranded DNA (ds-DNA) antibodies were detected using Crithidia luciliae indirect immunofluorescence test (EUROIMMUN, Lübeck, Germany). Anti-extractable nuclear antigen (ENA) antibodies, including anti-Sm, anti-SSA, anti-SSB, and anti-RNP antibodies, were detected using immunodotting assay (EUROIMMUN, Lübeck, Germany). Anti-cardiolipin antibodies and anti-*β*_2_GP-1 antibodies were detected using enzyme-linked immunosorbent assay (ELISA) (EUROIMMUN, Lübeck, Germany).

#### 2.3.1. Detection of ANCA

ANCA tests were performed by both indirect immunofluorescence (IIF) assay and antigen-specific enzyme-linked immunosorbent assay (ELISA). Standard IIF assay was performed using precooled ethanol fixed normal peripheral neutrophils as substrate according to the manufacturer (EUROIMMUN, Lübeck, Germany). The use of Hep-2 cell and paraformaldehyde-fixed neutrophils may allow the distinction between ANA and p-ANCA. In antigen-specific ELISA, two highly purified known ANCA antigens, PR3 and MPO, purified as previously reported [15] were used as solid-phase ligands.

#### 2.3.2. Quantification of Plasma Complement Components

Plasma concentrations of major human complement components were determined by enzyme-linked immunoassays, including complement fragments C5a (Quidel Corporation, San Diego, CA), C3a (Quidel Corporation, San Diego, CA), Bb (Quidel Corporation, San Diego, CA), soluble C5b-9 (*SC5b-9*, Quidel Corporation, San Diego, CA), properdin (Uscnk Life Science Inc., Wuhan, China), and C3 (Quidel Corporation, San Diego, CA). All the complement components were assayed according to the manufacturer's instructions. The principle of the assays was a four-step procedure: (I) microassay plates were precoated with murine monoclonal antibodies binding specifically to the complement components; (II) plasma samples were added according to the optimal dilutions, incubation time, and temperature from the instructions; (III) horseradish peroxidase conjugated antibodies binding to the complement components adsorbed on the plates were added; (IV) chromogenic substrate was added to determine the concentration of components.

The methods to detect plasma C4BP, C1q, and MBL (mannan-binding lectin) were the same as previously described with mild modification [16–18].

### 2.4. Renal Histopathology

The renal biopsy specimens were examined by light microscopy, direct immunofluorescence, and electron microscopy techniques.

#### 2.4.1. Light Microscopy Examination

Renal biopsy specimens were fixed in 4.5% buffered formaldehyde for light microscopy. Consecutive serial 3 *μ*m sections were used for histological staining. Stains employed included haematoxylin and eosin (H&E), periodic acid-Schiff (PAS), silver methenamine (Meth), and Masson's trichrome.

Crescentic glomerulonephritis was defined as over half of the total glomeruli affected by large crescents (the crescent takes up over half space in Bowman's capsule) by light microscope, which should be included in class IV-G lupus nephritis [5].

Renal thrombotic microangiopathy (TMA) was characterized by interlobular artery, arteriole, and glomerular capillary lesions, including endothelial cell swelling, lumen narrowing or obliteration, and thrombi formation by light microscopy. Swelling of glomerular endothelial cells, detachment from the glomerular basement membrane, and widening of the subendothelial space were identified by electron microscopy [19].

Podocytopathy was defined as podocyte effacement. Biopsy findings revealed either no glomerular immune deposits or sparse deposits, which were confined to the glomerular mesangium. The characteristic pathological glomerular abnormality was ultrastructural and resided in the visceral glomerular epithelial cells. The glomerular lesions included idiopathic minimal change glomerulopathy and focal and segmental glomerulosclerosis [3, 20].

Pathological parameters such as activity indices (AI) and chronicity indices (CI) were approached by renal pathologists using a modification of a previously reported system involving semiquantitative scoring of specific biopsy features [21, 22].

#### 2.4.2. Direct Immunofluorescence Examination

Direct immunofluorescence for immunoglobulin G (IgG), immunoglobulin A (IgA), immunoglobulin M (IgM), C3, C1q, and fibrin deposits was semiquantitatively graded from 0 to 4 according to the intensity of fluorescence. The glomeruli with sclerosis were excluded.

#### 2.4.3. Electron Microscopy Examination

Renal biopsy specimens were fixed in 2.5% paraformaldehyde for electron microscopy. After being embedded in epon, ultrathin sections were mounted on metal grids and stained with uranyl acetate before being viewed in a transmission electron microscope (JEM-1230; JEOL, Tokyo, Japan).

### 2.5. Blood Samples

For detection of ANCA and complement, plasma samples were obtained from peripheral blood at the same day of renal biopsy before initiation of immunosuppressive treatment. The blood samples of patients and controls were drawn into EDTA tubes. The plasma was collected immediately by centrifugation at 2000 g for 15 min at 4°C. All plasma samples were stored at −80°C until use. Repeated freeze/thaw cycles were avoided.

Informed consent was obtained for blood sampling and renal biopsy from each patient. The research was in compliance of the Declaration of Helsinki. The design of this work was approved by the local ethical committees.

### 2.6. Statistical Analysis

Statistical software SPSS 16.0 (SPSS, Chicago, IL, USA) was employed for statistical analysis. Quantitative data were expressed as mean ± SD, and median with range (minimum, maximum). For comparison of clinical and pathological features of patients, Student's *t*-test, one-way ANOVA analysis of variance, and Chi-square test were used. Kaplan-Meier curves were used to analyze patients' prognosis. Survival analysis was performed using the log-rank test. Results were expressed as hazard ratio (HR) with 95% confidence intervals (CI). Statistical significance was considered as *P* < 0.05.

## 3. Results

### 3.1. General Data of Patients with Scanty Immune Deposits Lupus Nephritis

**Figure 1:**
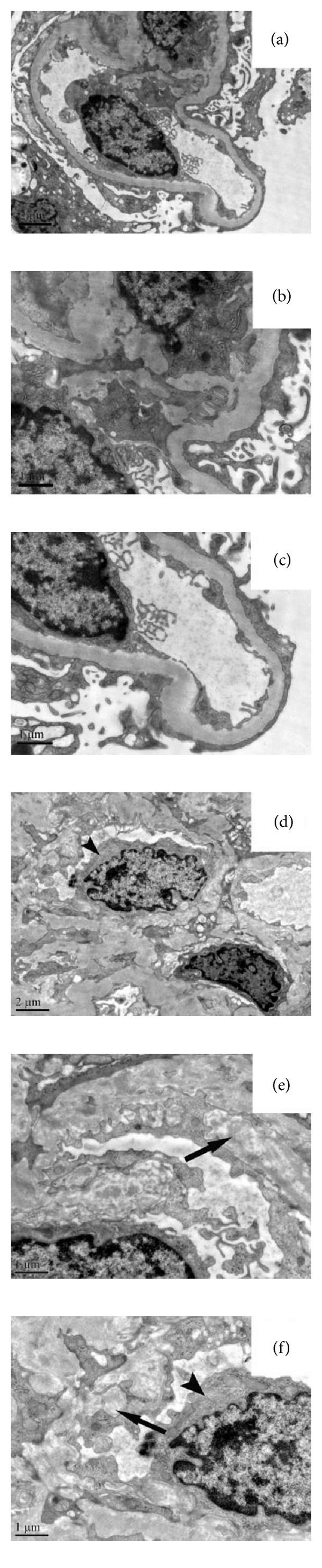
Electron micrographs of cases with scanty immune deposits lupus nephritis. (a)–(c) showed one case of mesangial proliferative lupus nephritis. No electron dense deposits were seen in mesangial area and glomerular basement membrane. Diffuse effacement of foot processes was observed. (d)–(f) showed one case of diffuse proliferative lupus nephritis combined with renal thrombotic microangiopathy. Glomerular endothelial cell (black pointer) was swollen, with increased mesangial matrix (d). Severe widening of subendothelial space (black arrow) with fluffy material and irregular cell projections; few of electron dense deposits were identified at higher magnification ((e), (f)). ((a), (d), original mag. ×10000) ((b), (c), (e), and (f), original mag. ×20000).

Among the 316 lupus nephritis patients enrolled in the study, 40 cases (12.66%) met the pathological criteria of scanty immune deposits nephritis, which were confirmed by electron microscopy (Figure 1).

In the scanty immune deposits group, 6 were male and 34 were female, with an average age of 39.78 ± 12.90 years at presentation. The majority of the patients (80%) were with hematuria. Half of the patients were with leukocyturia and 62.5% with nephrotic syndrome. The median amount of urine protein was 4.04 g/24 hours. The median value of serum creatinine was 102 mmol/dL (80.25–189.50 mmol/dL) upon diagnosis. The mean level of SLEDAI was 17.92 ± 5.45.

According to the 2003 classification of lupus nephritis, 6 patients were classified as class II (15%, including 1 case combined with minimal change disease), 9 cases as class III (22.5%), and 25 cases as class IV (62.5%, including 1 case combined with TMA and 7 cases with crescentic glomerulonephritis).

All of the patients received oral prednisone therapy. The majority of patients completed treatment with oral cyclophosphamide (3/40) or monthly intravenous cyclophosphamide (600–800 mg/month) (20/40). The other patients received mycophenolate mofetil (3/40), leflunomide (8/40), and azathioprine (4/40). Two patients received prednisone alone. Twenty-six patients achieved clinical remission, 8 with complete remission and 18 with partial remission. Fourteen patients presented with treatment failure.

We further compared the clinical and pathological characteristics of scanty immune deposits and immune complex deposits patients with lupus nephritis.

### 3.2. Comparison of Clinical and Laboratory Parameters between Patients with Scanty Immune Deposits and Immune Complex Deposits Lupus Nephritis

The clinical and laboratory features of patients in the two groups were listed in Tables 1 and 2.

The average age was significantly older in scanty immune deposits group than that in immune complex deposits group (*P* < 0.001). There were no significant differences between the two groups in other clinical indices.

In laboratory findings, there were significantly lower hemoglobin level (*P* = 0.009) and higher value of serum creatinine (*P* = 0.01) in scanty immune deposits group than those in immune complex deposits group.

Twenty-one out of the 40 patients (52.5%) in scanty immune deposits group were ANCA positive including 15 with p-ANCA and 2 with c-ANCA by IIF, 10 with anti-MPO antibodies, and 1 with anti-PR3 antibodies by ELISA. 28 out of the 276 patients (10.1%) in immune complex deposits group were ANCA positive including 20 with p-ANCA by IIF and 9 with anti-MPO by ELISA. The difference was significant (*P* < 0.001).

### 3.3. Comparison of Plasma Complement Components Levels between Patients with Scanty Immune Deposits And Immune Complex Deposits Lupus Nephritis

The levels of plasma complement components of patients in the two groups were listed in Table 3.

The normal levels of plasma MBL, C3a, C5a, and soluble C5b-9 were 1532 ± 1020 ng/mL, 100.87 ± 70.55 ng/mL, 9.32 ± 7.88 ng/mL, and 467.41 ± 545.23 ng/mL, respectively. They were significantly higher in patients with scanty immune deposits and immune complex deposits lupus nephritis than those in normal controls (*P* < 0.01, *P* < 0.01; *P* < 0.01, *P* < 0.01; *P* < 0.01, *P* < 0.01; *P* < 0.01, *P* < 0.01, resp.).

The normal levels of plasma C1q, properdin, Bb, C4BP, factor H, and C3 were 61.96 ± 10.50 *μ*g/mL, 22.58 ± 9.67 *μ*g/mL, 0.69  ±  0.45 *μ*g/mL, 326.59 ± 87.25 *μ*g/mL, 515.04 ± 134.08 *μ*g/mL, and 0.80 ± 0.17 mg/mL, respectively. They were significantly lower in patients with scanty immune deposits and immune complex deposits lupus nephritis than those in normal controls (*P* < 0.01, *P* < 0.01; *P* < 0.01, *P* < 0.01; *P* < 0.01, *P* < 0.01; *P* < 0.01, *P* < 0.01; *P* < 0.01, *P* < 0.01; *P* < 0.01, *P* < 0.01, resp.).

#### 3.3.1. Plasma Levels of C1q

C1q is the first component in the classical pathway of complement activation. The level of C1q was significantly higher in patients with scanty immune deposits lupus nephritis than that in immune complex deposits lupus nephritis (34.78 ± 5.65 *μ*g/mL versus 22.17 ± 3.08 *μ*g/mL, *P* = 0.005).

#### 3.3.2. Plasma Levels of MBL

MBL serves as a trigger for the activation of lectin pathway. There was no significant difference in MBL levels between patients with scanty immune deposits and immune complex deposits lupus nephritis (*P* = 0.675).

#### 3.3.3. Plasma Levels of C4BP

C4BP is the main fluid-phase inhibitor of the complement activation. It exerts inhibitory function by enhancing the decay of classical/lectin pathway C3 convertase, C4b2a [23], as well as the alternative pathway C3 convertase, C3bBb [24].

There was no significant difference in C4BP levels between patients with scanty immune deposits and immune complex deposits lupus nephritis (*P* = 0.389).

#### 3.3.4. Plasma Levels of Bb, Properdin, and Factor H

Properdin is critical in the stabilization of alternative pathway C3 convertase. Bb is an activation fragment of factor B in the alternative complement pathway. Complement factor H is an abundant plasma complement regulator that inhibits alternative pathway activation by inhibiting the formation and accelerating the decay of C3 convertase and acting as a complement factor I cofactor, which inactivates C3b to iC3b. Measurement of properdin, Bb, and factor H in plasma provided evidence for the activation of the alternative complement pathway [25–27].

The level of Bb was significantly higher in patients with scanty immune deposits lupus nephritis than that in immune complex deposits lupus nephritis (1.81; 0.62–2.54 *μ*g/mL versus 1.01; 7–1.74 *μ*g/mL, *P* = 0.02). The level of factor H was significantly lower in patients with scanty immune deposits lupus nephritis than that in immune complex deposits lupus nephritis (302.19 ± 110.47 *μ*g/mL versus 407.52 ± 181.44 *μ*g/mL, *P* = 0.003). There was no significant difference in properdin level between patients with scanty immune deposits lupus nephritis and immune complex deposits lupus nephritis (*P* = 0.357).

#### 3.3.5. Plasma Levels of C3, C3a, C5a, and SC5b-9

Activation of complement results in the conversion of C3 to C3a and C3b and then the formation of a C5 convertase multimolecular enzyme capable of cleaving C5 to C5a and C5b. The terminal complement complex (C5b-9) is generated by the assembly of C5b through C9 as a consequence of activation of complement system. Therefore, we tested plasma C3, C3a, C5a, and soluble C5b-9 (*SC5b-9*) levels to reflect total complement activation in circulation.

There were no significant differences in plasma concentration of C3, C3a, C5a, and *SC5b-9* between patients with scanty immune deposits and immune complex deposits lupus nephritis (*P* = 0.541, *P* = 0.134, *P* = 0.446, and *P* = 0.227, resp.).

### 3.4. Comparison of Renal Histopathologic Parameters between Patients with Scanty Immune Deposits and Immune Complex Deposits Lupus Nephritis

The renal histopathological features of patients with and without immune deposits lupus nephritis were listed in Table 4.

In comparison with immune deposits group, patients with scanty immune deposits group had significantly higher scores of cellular crescents (*P* = 0.015). There were no significant differences in other pathological indices between the two groups. The ratios of thrombotic microangiopathy, crescentic glomerulonephritis, and podocytopathy were not significantly different between the two groups (2.17% versus 8.33%, *P* = 0.242; 15.22% versus 9.78%, *P* = 0.052; 2.17% versus 0, *P* = 0.307, resp.).

### 3.5. Comparison of Treatment and Outcomes between Patients with Scanty Immune Deposits and Immune Complex Deposits Lupus Nephritis

The treatment and outcomes of patients with and without immune deposits lupus nephritis were detailed in Table 5. There was no significant difference in treatment algorithm between the two groups. The rates of complete remission and partial remission were not significantly different. The incidence of treatment failure was significantly higher in scanty immune deposits group than that in immune complex deposits group (36.95% versus 19.20%, *P* = 0.007).

During a similar follow-up time (average for nearly 5 years), the renal relapse rate was similar (13.79% versus 12.56%, *P* = 0.819) between the two groups.

**Figure 2:**
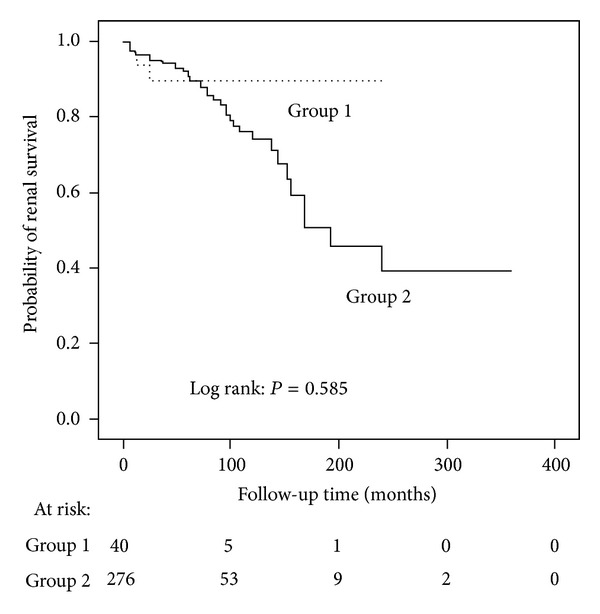
Comparison of renal outcomes between patients with scanty immune deposits (Group 1) and immune complex deposits lupus nephritis (Group 2).

Regarding long-term survival, there were no significant differences in mortality and renal outcomes between scanty immune deposits and immune deposits groups (*P* = 0.598, *P* = 0.585, resp., Figure 2). In scanty immune deposits group, one patient died due to infection; 6 patients reached the secondary end point including 1 with doubling of serum creatinine and 5 with ESRD. In immune complex deposits group, 2 patients died due to heart failure and cerebral hemorrhage, respectively; 35 patients reached the secondary end point, all with ESRD.

**Figure 3:**
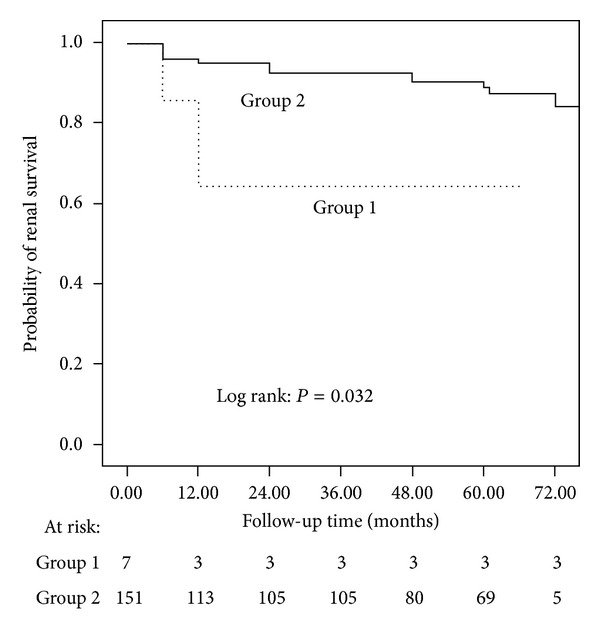
Comparison of renal outcomes between patients with scanty immune deposits crescentic lupus nephritis (Group 1) and immune complex deposits class IV lupus nephritis (Group 2).

We further compared renal outcomes between the scanty immune deposits class III lupus nephritis patients with those of immune complex deposits class III lupus nephritis and between scanty immune deposits crescentic lupus nephritis and immune complex deposits class IV lupus nephritis. In scanty immune deposits class III lupus nephritis group, 0 of the 9 patients reached the secondary end point. In immune complex deposits class III lupus nephritis group, one of the 40 patients reached the secondary end point. The ratio did not reach significant difference. In scanty immune deposits crescentic lupus nephritis group, 2 of the 7 patients reached the secondary end point. In immune complex deposits class IV lupus nephritis group, 30 of the 151 patients reached the secondary end point. Survival analysis showed that scanty immune deposits crescentic group had significantly worse renal outcome than that in immune complex deposits class IV group (*P* = 0.032, Figure 3).

Using the log-rank test for univariate survival analysis of renal prognosis in all the patients with lupus nephritis, we found that scanty immune deposits nephritis was not a risk factor for renal outcome in lupus nephritis. Other univariate risk factors included serum creatinine value, hemoglobin value, anti-SSB antibody, total activity indices score, cellular crescents, interstitial inflammatory cell infiltration, total chronicity indices score, fibrous crescents, and interstitial fibrosis (see Table 6 for details).

## 4. Discussion

It is well documented that various immunoglobulins deposited together with complements were found in the affected glomeruli in lupus nephritis. Accordingly, lupus nephritis was considered to be the classical type of immune complex associated glomerulonephritis. It is rare for lupus nephritis to contain no immune complex deposits, although there have been several cases of scanty immune deposits lupus nephritis reported previously [1–3, 28–30]. The clinicopathological features, outcomes, and possible pathogenesis of scanty immune deposits lupus nephritis should be studied.

The data arising from our study showed that the scanty immune deposits lupus nephritis was not uncommon, which accounted for 13% of all the lupus nephritis in our center. Of course, the glomeruli under pathological detection with sclerosis were excluded, and all the patients did not accept immunosuppressive treatment when the renal biopsies were done, which excluded the influences of therapy on the immune complex deposits in kidneys in our study. Previous studies indicated that the most possible explanation of scanty immune deposits lupus nephritis was that it might overlap with other scanty immune deposits-immune diseases, such as TMA [1], true renal vasculitis [29], ANCA-associated necrotizing and crescentic glomerulonephritis [2], glomerular podocytopathy [3], and glomerular lesions in kidney transplants [28]. Thus, we firstly focus on the presences of above disease in the scanty immune deposits group. We found that there were one patient with minimal change disease, one patient with TMA, and seven patients with true crescentic glomerulonephritis based on the strict diagnostic criteria [3, 5, 9]. But there was no significant difference in the ratios of the three pathological changes between scanty immune deposits group and immune complex deposits group.

After further comparisons of clinical, laboratory, and pathological features between the two groups, we found that the patients with scanty immune deposits lupus nephritis presented with older age, higher value of serum creatinine, and lower value of hemoglobin. Furthermore, significantly higher score of cellular crescents was found in scanty immune deposits group compared with immune complex deposits group evaluated by NIH scoring system. More importantly, by immunofluorescence assay and ELISA, we found that the positive ratio of ANCA was significantly higher in scanty immune deposits group than that in immune complex deposits group. Our previous study also showed that crescentic lupus nephritis presented with lower intensity of immunoglobulins and higher ratio of ANCA [31]. Thus, it highlights the importance of ANCA in the pathogenesis of scanty immune deposits crescentic lupus nephritis.

ANCA, as one of autoantibodies in SLE, might be implicated as a pathogenic factor for the development of scanty immune deposits crescentic lupus nephritis. Nasr et al. recently proposed that one of the two conditions (ANCA and lupus nephritis) may be creating fertile conditions for the second to develop [32]. It was suggested that lupus nephritis might facilitate the process of MPO autoantibody formation by promoting neutrophil degranulation and priming neutrophils to increase surface expression of MPO. Besides, the presence of ANCA was most closely associated with vasculitic lesions and the typical characteristic of ANCA-associated renal vasculitis was scanty immune deposits. So, it was suggested that the presence of ANCA in patients with SLE might indicate overlaps of SLE and ANCA-associated vasculitis [33], especially for those with the disproportionate necrotizing and crescentic features in lupus nephritis [31].

On the other hand, over half of the patients with scanty immune deposits lupus nephritis in our study were with ANCA negative. Apart from ANCA, other mechanisms of developing scanty immune deposits lupus nephritis were thought of. The role of complement in SLE was important and it was closely associated with immune complex formation. So, we further detected the plasma complement components levels, including classical, alternative, and mannose-binding lectin (MBL) pathways in our patients with lupus nephritis. The results showed that there were significantly higher levels of C1q and Bb and lower level of factor H in scanty immune deposits group compared with immune complex deposits group, which indicated the predominant activation of the complement alternative pathway in the pathogenesis of scanty immune deposits lupus nephritis.

The role of complement activation played in SLE and lupus nephritis has been assumed for many years. It was acknowledged that immune complexes formed by self-antigens and autoantibodies might activate the classical pathway, generating inflammatory mediators and resulting in tissue damage. However, convincing evidences indicated that the alternative pathway was also activated and participated in the pathogenesis of SLE, especially in lupus nephritis [34–39]. The analysis of complement components in our study showed that all the three pathways might be activated and involved in the pathogenesis of lupus nephritis. However, the higher levels of C1q and Bb indicated that alternative pathway activation, other than classical pathway, was more notable in scanty immune deposits lupus nephritis than that in immune complex deposits group. In addition, lower level of factor H, an important inhibitory factor in alternative pathway by accelerating the decay of the alternative pathway C3-convertase (C3bBb), also supported that there might exist overactivation of alternative pathway. A recent study even showed that factor H deficiency could accelerate the development of lupus nephritis in lupus-prone mice MRL-lpr [40].

Interestingly, recent studies, including the mouse model of anti-MPO IgG mediated glomerulonephritis and human ANCA associated vasculitis, suggested that complement alternative pathway activation was crucial for ANCA associated vasculitis development [41–43]. Taken together, ANCA and complement alternative pathway activation might both be involved in the development of scanty immune deposits lupus nephritis which need further investigation.

There is no well-established guideline for the treatment of scanty immune deposits lupus nephritis. The therapy between scanty immune deposits and immune complex deposits groups in our patients was similar, both including immunosuppressive treatment. Although the ratio of renal remission rate was not different in the two groups, higher proportion of treatment failure was found in scanty immune deposits group. It might be related to the more severe clinical and pathological characteristics in scanty immune deposits group.

The 5-year survival rate and renal outcome between the two groups were similar. Scanty immune deposits nephritis was not a risk factor for renal outcome by log-rank test for univariate survival analysis of renal prognosis. However, as scanty immune deposits lupus nephritis consisted of a group of pathological types, we further compared renal outcomes between different subgroups. Interestingly, we found that scanty immune deposits crescentic group had significantly worse renal outcome than that in immune complex deposits class IV group, which needs further observation.

There were some limitations in this study: (I) despite being a large cohort study, it was a retrospective analysis; (II) the staining of complement components in kidney tissues, especially in alternative pathway, is important; (III) longer duration of followup and multicenter study are needed.

In conclusion, scanty immune deposits lupus nephritis was not uncommon. Patients with scanty immune deposits lupus nephritis had more severe kidney damage. ANCA-associated vasculitis and activation of complement alternative pathway might be involved in the pathogenesis of scanty immune deposits lupus nephritis.

## Figures and Tables

**Table 1:** Comparison of clinical data between patients with scanty immune deposits and immune complex deposits lupus nephritis.

	Scanty immune deposits	Immune complex deposits	*P* value
Number of patients	40	276	
Age (mean ± SD) (years)	39.78 ± 12.90	32.06 ± 10.94	<*0.001 *
Gender (male/female)	6/34	42/234	0.971
Number with fever (noninfection) (%)	11 (27.5)	82 (29.7)	0.774
Number with malar rash (%)	17 (42.5)	149 (54.0)	0.174
Number with photosensitivity (%)	6 (15.0)	57 (20.7)	0.403
Number with alopecia (%)	14 (35.0)	84 (30.4)	0.560
Number with oral ulcer (%)	11 (23.9)	83 (30.1)	0.739
Number with arthritis (%)	25 (62.5)	143 (51.8)	0.205
Number with serositis (%)	5 (12.5)	46 (16.7)	0.503
Number with neurologic disorder (%)	3 (7.5)	23 (8.3)	0.858
Number with anemia (%)	31 (77.5)	183 (66.5)	0.165
Number with leukocytopenia (%)	23 (57.5)	123 (44.6)	0.125
Number with thrombocytopenia (%)	10 (25.0)	93 (33.8)	0.267
Number with hematuria (%)	32 (80.0)	210 (76.1)	0.585
Number with leukocyturia (noninfection) (%)	23 (57.5)	145 (52.5)	0.557
Number with nephrotic syndrome (%)	25 (62.5)	161 (58.8)	0.653
SLEDAI (median; interquartile range)	18, 13–23	17, 14–21	0.751

*P* < 0.05, italic values refer to the significant value between the two groups.

**Table 2:** Comparison of laboratory data between patients with scanty immune deposits and immune complex deposits lupus nephritis.

	Scanty immune deposits	Immune complex deposits	*P* value
Number of patients	40	276	
Hemoglobin (g/L) (mean ± SD)	91.18 ± 24.39	102.16 ± 24.93	*0.009 *
Urine protein (g/24 hours) (median; interquartile range)	4.04, 1.96–5.36	4.34, 2.20–7.07	0.348
Serum creatinine (mmol/dL) (median; interquartile range)	102, 80.25–189.50	81, 67–126	*0.01 *
Number with positive ANA (%)	39 (97.5)	273 (98.9)	1
Number with positive anti-dsDNA (%)	25 (64.1)	191 (69.2)	0.521
Number with positive anti-Sm (%)	13 (33.3)	69 (25.0)	0.267
Number with positive anti-SSA (%)	16 (41.0)	124 (44.9)	0.646
Number with positive anti-SSB (%)	3 (7.9)	32 (11.6)	0.493
Number with positive anti-RNP (%)	12 (30.8)	84 (30.4)	0.966
Number with positive anticardiolipin (%)	2 (2/34, 6.9%)	17 (17/223, 7.6%)	1
Number with positive anti-*β*2 GP-1 (%)	4 (4/34, 6.9%)	15 (15/223, 6.7%)	1
Number with positive ANCA (%)	21 (52.5)	28 (10.1)	<*0.001 *

*P* < 0.05, italic values refer to the significant value between the two groups.

**Table 3:** Comparison of plasma complement components levels between patients with scanty immune deposits and immune complex deposits lupus nephritis.

	Scanty immune deposits	Immune complex deposits	*P* value	Normal range
Number of patients	40	276		
C1q (ug/mL) (mean ± SD)	34.78 ± 5.65	22.17 ± 3.08	*0.005 *	61.96 ± 10.50
MBL (ng/mL) (median; interquartile range)	2223 575–3719	1768 476–2879	0.663	1532 ± 1020
Properdin (ug/mL) (mean ± SD)	13.26 ± 6.32	15.39 ± 6.05	0.379	22.58 ± 9.67
Bb (ug/mL) (median; interquartile range)	1.81 0.62–2.54	1.01 0.7–1.74	*0.02 *	0.69 ± 0.45
C4BP (ug/mL) (mean ± SD)	243.23 ± 131.54	221.56 ± 79.25	0.262	326.59 ± 87.25
Factor H (ug/mL) (mean ± SD)	302.19 ± 110.47	407.52 ± 181.44	*0.002 *	515.04 ± 134.08
C3 (mg/mL) (median; interquartile range)	0.58 0.38–1.22	0.54 0.31–0.81	0.671	0.80 ± 0.17
C3a (ng/mL) (median; interquartile range)	4.09 1.5–801.27	3.15 1–562.32	0.321	100.87 ± 70.55
C5a (ng/mL) (median; interquartile range)	20.32 11.26–30.79	17.25 10.41–29.33	0.454	9.32 ± 7.88
Soluble C5b-9 (ng/mL) (median; interquartile range)	3.00 1–754.35	4.10 1–698.41	0.239	467.41 ± 545.23

*P* < 0.05, italic values refer to the significant value between the two groups.

**Table 4:** Comparison of renal pathological data between patients with scanty immune deposits and immune complex deposits lupus nephritis.

	Scanty immune deposits	Immune complex deposits	*P* value
Number of biopsies	40	276	
Class II (%)	6 (15)	13 (4.71)	0.195
Class III (%)	9 (22.5)	46 (16.67)	
Class IV (%)	25 (62.5)	151 (54.7)	
Class V (%)	0 (0)	64 (23.2)	
Class VI (%)	0 (0)	2 (0.72)	
AI score (mean ± SD)	8.70 ± 4.58	7.50 ± 4.65	0.128
Endocapillary hypercellularity (median; interquartile range)	3, 1–3	3, 1–3	0.482
Cellular crescents (median; interquartile range)	2, 0–4	0, 0–2	*0.015 *
Karyorrhexis/fibrinoid necrosis (median; interquartile range)	0, 0–2	0, 0–2	0.687
Subendothelial hyaline deposits (median; interquartile range)	1, 0–2	1, 0–2	0.913
Interstitial inflammation (median; interquartile range)	1, 1-2	1.1–1	0.067
Glomerular leukocyte infiltration (median; interquartile range)	1, 0-1	1, 0-1	0.694
CI score (mean ± SD)	3.10 ± 2.29	2.75 ± 1.99	0.31
Glomerular sclerosis (median; interquartile range)	0, 0-1	0, 0-1	0.717
Fibrous crescents (median; interquartile range)	0.0-0	0, 0-0	0.795
Tubular atrophy (median; interquartile range)	1, 1-2	1, 1-1	0.09
Interstitial fibrosis (median: interquartile range)	1, 1–1.75	1, 1-1	0.182

*P* < 0.05, italic values refer to the significant value between the two groups.

**Table 5:** Comparison of treatment between patients with scanty immune deposits and immune complex deposits lupus nephritis.

	Scanty immune deposits	Immune complex deposits	*P* value
Number of patients (%)	40	276	
Treatment			
P	40 (100)	276 (100)	1
CYC	23 (57.50)	156 (56.52)	0.907
AZA	4 (10.00)	21 (7.60)	0.833
MMF	3 (7.50)	17 (6.15)	1
LEF	8 (20.00)	31 (11.23)	0.187
Treatment response			
CR	8 (20.00)	78 (28.26)	0.560
PR	18 (45.00)	145 (52.54)	0.373
TF	14 (35)	53 (19.20)	*0.001 *
Duration of followup (months)	38, 6–78	48, 8.5–84	0.952

Relapse rate	4 (4/26, 15.38%, 3 with nephritic relapse and 1 with proteinuric relapse)	28 (28/223, 12.56%, 20 with nephritic relapse and 8 with proteinuric relapse)	0.922

Note: P: oral prednisone; CYC: cyclophosphamide; AZA: azathioprine; MMF: mycophenolate mofetil; LEF: leflunomide; CR: complete remission; PR: partial remission; TF: treatment failure.

*P* < 0.05, italic values refer to the significant value between the two groups.

**Table 6:** Univariate survival analysis of patients renal prognosis with lupus nephritis.

	HR	95% confidence interval		*P* value
Age	0.240	0.056	1.031	0.055
Sex	0.514	0.158	1.674	0.269
C3	0.693	0.167	2.876	0.613
Proteinuria	0.948	0.226	3.971	0.942
Serum creatinine value	16.063	6.746	38.251	<*0.001 *
Hemoglobin	0.285	0.154	0.530	<*0.001 *
ANA	0.389	0.053	2.855	0.353
Anti-ds-DNA antibody	1.376	0.710	2.670	0.345
Anti-Sm antibody	0.972	0.476	1.984	0.938
Anti-SSA antibody	0.786	0.417	1.479	0.455
Anti-SSB antibody	2.878	1.365	6.067	*0.005 *
Anti-RNP antibody	0.721	0.361	1.441	0.355
Anticardiolipin antibody	1.175	0.350	3.939	0.794
SLEDAI	0.933	0.255	4.430	0.933
Activity indices (AI) score	3.941	1.876	8.279	<*0.001 *
Endocapillary hypercellularity	1.814	0.803	4.099	0.152
Cellular crescents	3.339	1.703	6.547	<*0.001 *
Karyorrhexis/fibrinoid necrosis	1.646	0.890	3.043	0.112
Subendothelial hyaline deposits	0.848	0.445	1.616	0.721
Interstitial inflammatory cell infiltration	5.492	2.859	10.547	<*0.001 *
Glomerular leukocyte infiltration	1.145	0.613	2.140	0.671
Chronicity indices (CIs) score	1.379	1.230	1.545	<*0.001 *
Glomerular sclerosis	1.025	0.243	4.320	0.793
Fibrous crescents	3.412	1.839	6.328	<*0.001 *
Tubular atrophy	25.129	0.497	1.271	0.107
Interstitial fibrosis	9.222	1.268	67.070	*0.028 *
Scanty immune deposits or immune complex deposits	**1.320**	**0.519**	**3.359**	**0.560**

*P* < 0.05, italic values refer to the significant value between the two groups.

Bold values refers to that scanty immune deposits nephritis was not a risk factor for a renal outcome in lupus nephritis.
